# The Possibility of Improving the Range and Quality of Air-to-Ground WebRTC-Based IoT Communication in Emergency Situations by Employing an External Transceiver

**DOI:** 10.3390/s24206533

**Published:** 2024-10-10

**Authors:** Krzysztof Wajda, Agnieszka Chodorek, Robert Ryszard Chodorek

**Affiliations:** 1 Institute of Telecommunications, Faculty of Computer Science, Electronics and Telecommunications, AGH University of Krakow, Al. Mickiewicza 30, 30-059 Krakow, Poland; kwajda@agh.edu.pl; 2 Department of Applied Computer Science, Faculty of Electrical Engineering, Automatic Control and Computer Science, Kielce University of Technology, Al. 1000-lecia P.P. 7, 25-314 Kielce, Poland; a.chodorek@tu.kielce.pl

**Keywords:** beamforming, internet of things, multimedia, real-time transmissions, unmanned aerial vehicle, WebRTC

## Abstract

The proliferation of new services, either interpersonal or machine-oriented, has generated new demands concerning the flexibility and efficiency of transmission. The ubiquity of multimedia communication in the current internet is seamlessly and successfully supported by the WebRTC concept. This paper reports on the study of the usage of a solution employing a proxy transmission unit for air-to-ground delivery of video streaming multiplexed with sensor data in the UAV-IoT system when using the WebRTC protocol stack. The comparative experiments were carried out for two cases employing the 802.11ac network with WebRTC: the first scenario (S1) without an external transceiver and the second scenario (S2) with an external transceiver working as a proxy of the ground receiver. The presented results compare the transmission conditions without (scenario S1) and with (scenario S2) the external transceiver in terms of the RSSI, the available data rate, and total throughput of transmission of multimedia data (video stream from the UAV camera and bursty data coming from employed sensors. The usefulness of the external transceiver used in a wide range of transmission conditions is clearly proven.

## 1. Introduction

The US Federal Aviation Administration (FAA) in its forecast for 2022–2042 considered the Unmanned Aerial Vehicles (UAV) sector to be enormously promising, including UAV applications in public services, such as conducting search and rescue support missions after disasters [1]. UAVs enable relatively quick, cheap and easy access to various locations and provide the ability to hover over a given location. This is particularly important in crisis or emergency situations, when reaching a given place by means other than air may be impossible or very difficult. As an effect, UAVs are used to monitor the endangered areas, deliver important shipments (e.g., medicines) and provide emergency communication in the disaster area [2,3]. In addition to operations during the event itself, UAVs are also used before a natural disaster (pre-disaster preparedness) as monitoring systems that implement the functionality of early warning systems (EWS) [3].

### 1.1. WebRTC Technology

Web Real-Time Communications (WebRTC) [4,5,6] is commonly used in popular Web browsers and supports multimedia acquisition and transport in a native way. Applications in WebRTC are written with HyperText Markup Language version 5 (HTML5) and JavaScript scripting language. Total settings for WebRTC are much more complex than in classic web applications since the sessions are described by using the Session Description Protocol (SDP) and established in JavaScript Session Establishment Protocol (JSEP). WebRTC applications are the equivalent of classic standalone multimedia applications managed using the Session Initiation Protocol (SIP). In the case of WebRTC, SIP has been replaced by JSEP in the management plane protocol stack, and this stack is similar to the SP-based one [7], used in standalone applications.

The quality of transmission is influenced by both the underlying network and the mechanisms of the transport layer. In the case of WebRTC, the transport of the media streams is conducted with the Real-time Transport Protocol (RTP), typically used in standalone multimedia applications, and the transport of non-media data with the Stream Control Transmission Protocol (SCTP). Both of these protocols operate over the User Datagram Protocol (UDP), and both provide Transmission Control Protocol (TCP)-friendly congestion control (i.e., TCP-like one). While RTP achieves TCP-friendliness by using TCP-Friendly Rate Control (TFRC) [8,9], SCTP directly implements the TCP congestion control algorithm. SCTP also implements the TCP error control [10]. Media streaming over RTP is not error-controlled, although transmission errors are reported to the sender.

RTP transmission reports are sent using the Real-time Transport Control Protocol (RTCP). The RTCP specification is an integral part of the RTP specification. Since RTP and SCTP transmissions are performed over UDP, which causes problems with firewalls and Network Address Translation (NAT) gateways in modern networks, the WebRTC control plane also includes a protocol suite for traversing firewalls and NATs, which consists of the Interactive Connectivity Establishment (ICE) protocol, the Session Traversal Utilities for NAT (STUN) protocol, and the Traversal Using Relay NAT (TURN) protocol. During transmission, media streams and non-media data flows are cryptographically protected. For this purpose, RTP uses the Secure Audio/Video Profile (RTP/SAVP), commonly called the Secure RTP (SRTP). SCTP packets are protected using the Datagram Transport Layer Security (DTLS) protocol.

The possibilities of native real-time communication in the web environment drew the attention of Internet of Things (IoT) system developers to WebRTC. WebRTC was used to transmit images from the camera and voice from the microphone, while data from sensors were transmitted using classic Web of Things (WoT) methods [11], and WebRTC was used to transmit data from web servers in WoT systems [12], as well. In another solution, data from lidars were transmitted via the WebRTC video channel, which was intended to provide real-time communication for time-sensitive applications [13]. The new WebRTC use cases postulated by the World Wide Web Consortium (W3C) include the IoT [14]. Transmitting data from sensors in a classic IoT way uses the ability of WebRTC for peer-to-peer transmission between web browsers, without the use of a web server [15].

The concept presented in [15] was implemented in our previous work, describing a flying parking monitoring system in which data from environmental sensors carried by a UAV were transmitted via SCTP, via the WebRTC web logical channel, and the WebRTC video channel was used to transmit information captured by the 4K camera [16]. The development version of this software has proven successful in prototypes of a pollution monitoring system [17] and a mobile weather station [18]. The experience from building these three systems was used to build the system presented in this paper.

### 1.2. Background and Related Work

In all UAV applications, an air-to-ground channel connecting the UAV to the remote ground station is crucial. The air-to-ground channel transmits UAV control data, telemetry data for UAV control (usually key UAV flight parameters, such as battery voltage, UAV position, speed, and altitude), and, last but not least, data from devices installed on board the UAV for a specific mission (e.g., cameras, sensors, routers). The most important solutions for wireless air-to-ground connections employ the Institute of Electrical and Electronics Engineers (IEEE) 802.11 standard [19,20,21,22,23,24,25], ZigBee standard [19], Worldwide Interoperability for Microwave Access (WiMAX) standard [19], Long Term Evolution (LTE) standard [19,21,26,27], the fifth-generation (5G) technology standard for broadband cellular networks [26,27,28,29] or proprietary solutions such as Shenzhen DJI Sciences and Technologies Ltd. (DJI) standards: OcuSync or Lightbridge [22,23,30].

The work [31] lists four factors affecting the range and quality of wireless transmissions. These are solutions used in the wireless network interface, antenna systems, radio interference and radio wave propagation. It follows that factors improving the range and quality of air-to-ground transmissions include an improved network interface [2,27] and an improved antenna system [32,33,34,35,36], which include the use of higher-gain omnidirectional antennas [21,37], directional antennas [32,33,34,36], Multiple Input Multiple Output (MIMO) antenna systems [38]. Multiple antennas also occur in beamforming [39,40,41,42,43,44], which exploits the interference between the signal emitted from each antenna segment. Thanks to appropriate signal processing techniques and steering of the antenna array, beamforming eliminates the interfering signal from a particular direction in such a way that at the required angles constructive interference is obtained, and at other angles, the interference is nullified by destructive interference [45]. As an effect, the main beam of the antenna is guided towards the desired direction, and the undesired beams are nullified at the transmitter and receiver [46]. Beamforming can be explicit or implicit [39], which means the receiver’ cooperation or lack thereof, respectively. Explicit beamforming transmitters and receivers exchange channel information, so this type of beamforming is able to better manage signal strength.

The radio wave propagation or, more precisely, rapid decrease in signal strength with distance is the basis for the idea of using an additional intermediate transmission unit, which can be placed on the ground [16,47,48] or in the air. Airborne nodes can be used both to improve the range and quality of air-to-ground transmission [21,34,38,49,50,51,52,53], and to improve properties of transmission between ground stations [34,37,54]. In both cases, intermediate nodes can also use other methods of range and quality improvement, such as external omnidirectional antennas with a higher gain [21,37] (here 5 dBi), or a directional antenna [34]. However, not all of these methods are suitable for working with highly mobile UAV-borne devices. As an example, the use of directional antennas for communication with highly mobile UAVs may require a tracking system [33,36].

Table 1 lists methods for improving the range and quality of IEEE 802.11 air-to-ground transmission between UAVs and ground stations, classified according to the factor presented in [31], the place where the method of improving the range and quality was implemented (air, ground, both air and ground), the basic concept of improving the range and quality, and the purpose of the solutions presented in the cited papers. Table 2 summarizes the IEEE standards used in each paper. In the cases summarized in Table 1, the methods used were based on all the factors mentioned in [31], from the wireless interface to propagation. The latter include various types of intermediate nodes: range extenders [48,49], nodes of mesh networks [21,49,50,51,52], nodes of chain networks [34,38] and an Extended Service Set (ESS) composed of access points (APs) connected through a wired Distribution System (DS) built as a 1 Gbps Ethernet network [47].

The solutions presented in Table 1 are both general-purpose ones and the ones for ensuring emergency communication. General purpose solutions are intended for typical tasks when the situation is static, or planned. Such tasks include, for example, flights over the same area. For this reason, one can find the use of antennas with vertical or horizontal polarization [35], beamforming [39,40,41,42,43], the use of chain network nodes transported by UAV [50,53], as well as extreme solutions: allowing only low mobility or, on the contrary, associated with highly mobile systems. An example of the first one is the use of directional antennas supported by automatic heading control of a UAV carrying a node of a chain network [34]. An example of the latter is the use of external antennas with higher gain [21,37] or the use of a mesh network [50].

Emergency communication solutions were intended for use in post-disaster emergency networks [38], for emergency communication in disaster areas [2,21,51], for search and rescue (SAR) purposes [52], and for both emergency communication in disaster and SAR [21,47,49]. By their nature, such solutions operate in dynamic, unusual and uncertain situations. Hence, the use of intermediate nodes dominates among emergency communication solutions, and UAV-borne network nodes are significantly overrepresented among them. This is due to the fact that the use of ground nodes requires access to the patrolled area and the ability to quickly supplement the missing infrastructure. The improvement of the range and quality of air-to-ground transmission in the case of ground access to the patrolled area is presented in [38]. In a situation such as that considered in [47], where the hypothetical area affected by the disaster was relatively small, the patrolled area may be inaccessible from the ground.

The use of an intermediate node shortens the critical section of the transmission path, which in the case of communication with UAVs is usually the air-to-ground section, while simultaneously extending and typically lengthening the path, which then consists of two (in the case of point-to-point transmission) or more (one more than without an additional node) segments. Any additional node involves additional costs, not only financial. The idea of using an intermediate node assumes, however, that despite increased costs, the shortening of the air-to-ground distance makes the cost/effectiveness ratio favorable. In the case of emergency communications based on UAV-borne network nodes, this assumption is questionable because the use of UAV-borne nodes dedicated to emergency communication will reduce the number of UAVs participating in disaster area monitoring or SAR tasks. The use of UAV-borne dual-purpose nodes, capable of carrying out monitoring tasks and at the same time providing emergency communications, will significantly reduce the amount of information that can be transmitted from a single flying monitoring system [52].

Table 3 summarizes the above considerations. Improving the range and quality of air-to-ground transmissions in situations where access to the patrolled area is impossible can be achieved both with and without the use of an intermediate node. This comes at a variable cost that includes more than just the cost of an additional node or the cost of replacing the network interface. In some solutions, the interface cannot be replaced and it is necessary to replace a large part of the system or even the entire system. Replacing the interface cannot be conducted on an ad hoc basis when needed so in the case of time-critical missions such as SAR, an additional, unacceptable cost is mission failure. An additional cost in the case of air nodes is both the cost of the carrier, i.e., the UAV, and the exclusion of carriers from performing SAR tasks. The cheapest way to improve the performance of air-to-ground transmissions is to use ground intermediate nodes [38,47], but then the area inaccessible from the ground cannot be too large [47].

There are also solutions that combine both methods discussed above. Typically, the improvement obtained using an intermediate node is combined with the improvement obtained without using an intermediate node. When both methods are combined the effects are also combined but so are the costs. In the hybrid method, the no-node improvement method is emulated with the ground-node method, which requires placing the node close to the ground station. The cost of the method is the cost of using the ground node. The hybrid method was used in the company’s solution [48], which used a range extender. The solution [48] is practically unavailable and not supported.It was abandoned in subsequent generations of drones, which use the OccuSync standard instead of 802.11. Where improved range is required, relays attached to the extension rod are currently used in their classic position [30], allowing for the transmission path to be divided into segments. For obvious reasons, they require access from the ground to the place where the rod can be located. The solution [48] does not support new versions of the 802.11 standard that are required in our experiments.

In this paper, an alternative hybrid solution was proposed that uses an access point that does not work in the range extender mode. To emphasize the unusual, ineffective location of the intermediate node and the need for the access point to meet conditions enabling effective improvement of range and quality despite such a location, this solution was called an external transceiver.

### 1.3. Motivations, Main Contributions and Organization of This Paper

The emergency communication solutions listed in Table 1 assumed the use of large hardware resources, and sometimes very large ones as in [52]. However, it cannot be guaranteed that such large resources will be available anytime and anywhere. As an effect, when resources are limited, another solution is needed. One that (1) is placed on the ground, because each available UAV may be needed to carry monitoring equipment; (2) cannot extend the range by dividing the critical section of the transmission path into two shorter sections because the accessibility of the disaster area cannot be guaranteed; (3) the increase in the range and quality must be implemented in a “seamless” way for the endsystems, as is the case of UAV-borne network nodes. As an effect, we propose to use a dedicated ground node, set close to the ground station. Such a node is unable to shorten the critical, air-to-ground section of the transmission path, so improving the transmission range and quality must be based on a factor mentioned in [31] other than propagation.

Such intermediate nodes must be able to transmit and receive simultaneously. It must be placed close to the receiver so that the signal strength drop between the node and the receiver is negligible. A node with these properties will be a proxy of the endsystem at the ground, so it will de facto be an element of the ground station. For this reason, we called it an external transceiver of a ground station or, for short, an external transceiver. Since the main area of operation of the external transceiver is over long air-to-ground distances, that is, where low signal strength occurs resulting in high error rates, the operation of the external transceiver cannot be considered in isolation from the operation of congestion control mechanisms. These mechanisms occur in the transport layer protocol, are triggered by transmission errors and adapted to the current network state based on the current packet error rate (PER). As an effect, PER will have a more significant impact on the throughput of air-to-ground transmission than if congestion control mechanisms were not present.

As was mentioned above, in our previous papers, we presented the prototype of a flying monitoring system based on WebRTC [16], which was then used for experiments assessing the possibility of using the existing network infrastructure to increase the range and quality of air-to-ground communications in the event of lack of access to the monitored area [47]. This paper focuses on the issue of increasing the range and quality of transmission in emergency situations, in the event of a lack of access to the monitored area and a lack of infrastructure, including the UAV-borne one, that could be used for this purpose. The main contributions of this paper are as follows:

- proposing the use of an external transceiver,
- conducting, in various network conditions, a number of field experiments related to the air-to-ground transmission of multiplexed video and sensor data in cases of using and not using an external transceiver,
- assessment of the improvement achieved by using an external transceiver made at three levels: network interface level (based on the increase in the received signal strength indicator, or RSSI), operating system level (based on the increase in available bit rate), application level (based on the increase in throughput).

The rest of the paper is organized as follows. Section 2 reports on the architecture of the flying monitoring system used in the experiments describes experiments carried out in the parking lot of AGH University of Krakow, and presents the measures of improvement used in the evaluation of the transmissions with and without the proxy transceiver. The results obtained from the field experiments are provided and commented on in Section 3. Section 4 summarizes obtained achievements and advocates for the usage of an external transceiver. Finally, Section 5 concludes our experiences.

## 2. Materials and Methods

This section describes the materials and methods used in the experiments and the further processing of the results. The first two subsections include the flying monitoring system that uses WebRTC for video and IoT data transmission, and which was the source of data transmitted air-to-ground in real-time, as well as the testbed, scenarios, and the course of the experiment. The last subsection describes the measurement of improvements that were used in this paper to assess the possibility and desirability of using external transceivers to increase the range and quality of air-to-ground WebRTC-based transmissions.

### 2.1. The Flying Monitoring System

As the flying monitoring system, the system described in [16] was used, which includes the air and ground stations. The air station consists of an IoT carrier, which is a quadcopter built on a 450 mm isosceles frame, and an IoT system built on a single board computer (SBC) Raspberry Pi 3 Model B+ (Raspberry Pi Foundation, Cambridge, United Kingdom). The Raspberry Pi is the most popular and powerful computer hardware platform used in IoT devices and one of the most energy-efficient SBCs. The Raspberry Pi version 3 has several built-in network interfaces, including 2.4 GHz and 5.0 GHz IEEE 802.11ac, as well as two Universal Serial Bus (USB) 3.0 ports and one 40-pin General Purpose Input-Output (GPIO) connector. In the air station, one of the USB 3.0 ports was used to connect a 4K camera, which is Manta MM9359FS (MANTA S.A., Warsaw, Poland), and the other was optionally used to connect an external IEEE 802.11ac network interface. The GPIO was used for connecting the positioning module, which is Waveshare SIM7000E (Waveshare International Limited, Shenzhen, China), and environmental sensors to the SBC. The 802.11ac standard was chosen because it is widely used in systems employing Raspberry Pi. This is due to the fact that the standard network interface in this SBC is 802.11ac and the difficulties in the availability of the market of newer (e.g., 802.11ax) network interfaces with a USB interface. Currently, the USB interface is the only possible one that allows an external network card to be connected to the Raspberry Pi.

The set of low-cost environmental sensors, used in experiments, includes a Bosch (Robert Bosch GmbH, Gerlingen, Germany) Sensortec BME280 (air pressure, temperature, and humidity sensor), a Measurement (TE Connectivity, Galway, Ireland) HTU21D (temperature and humidity sensor), a Silicon Laboratories (Silicon Laboratories Inc., Austin, TX, USA) SI1145 (UV index sensor), a Vishay Semiconductors (Vishay Intertechnology, Malvern, PA, USA) VEML6070 (UV radiation sensor), and a Waveshare MQ-135 (gas sensor). The weather sensors and the gas sensor were previously used in our prototypes of the flying monitoring system for pollution sensing [17] and the IoT system for mobile weather stations [18]. Since the sensor service of our WebRTC-based monitoring application is highly device-dependent, using the same types of sensors allowed us to reuse sensor-dependent code snippets written and extensively tested for previous systems.

**Figure 1:**
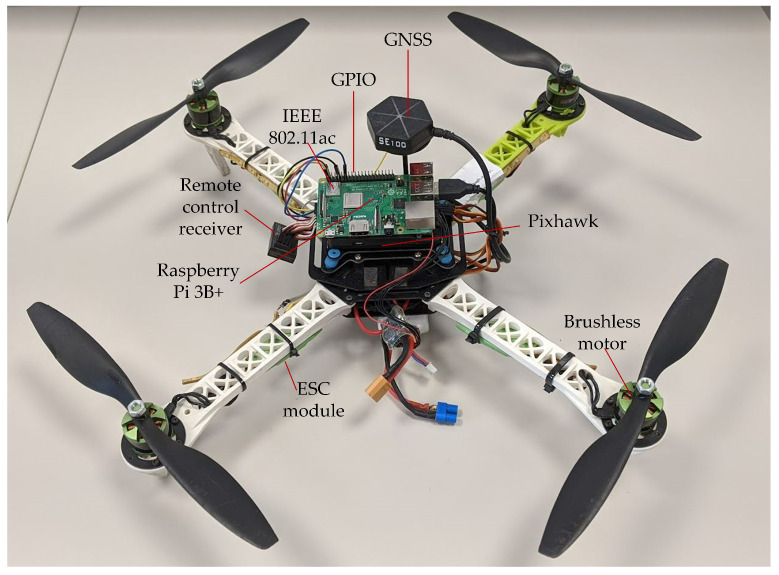
Air station.

An overview of the complete air station is shown in Figure 1. The Raspberry Pi is placed in the center of the air station. The rectangle in the upper left corner of the Raspberry Pi board, next to the 40-pin GPIO, is the IEEE 802.11ac network interface. The Global Navigation Satellite System (GNSS) UAV module is visible above the SBC (hexagonal device at the very top of the air station). The Pixhawk flight controller is placed under the SBC (partly hidden under the SBC, and partly visible in the shadow of the Raspberry Pi board). Both the SBC and the Pixhawk are attached to the UAV using rubber vibration dampers. Underneath there is a gimbal with the UAV camera (not visible in the picture). On the left hangs the UAV remote control receiver. Brushless motors with propellers are mounted at the ends of the frame arms. In the center and under each arm is an Electronic Speed Control (ESC) module that controls the speed of the respective electric motor.

On the SBC, the WebRTC-based monitoring application was run. This is a development version of the application presented in [16], which combines the functionality of an IoT broker and a media streaming application. It composes, manages and controls the real-time media stream and non-real-time data flow. The media stream is a congestion-controlled stream of video frames from the UAV camera. The non-real-time data flow is the congestion-controlled, reliable data flow consisting of Message Queue Telemetry Transport (MQTT) messages that convey sensor data and their spatio-temporal metadata. The real-time media stream and non-real-time data flow are multiplexed by WebRTC and sent air-to-ground as an aggregated stream of heterogeneous data. If the transmission is error-free, that is, if the throughput of the aggregated stream is not limited by congestion control, then the total throughput of the aggregated stream is 20 Mbps.

Both video and sensor data are sent through the production network to the ground, where they are received by a monitoring application running on a WebRTC multimedia and monitoring station (WMMS). WMMS is a part of the ground station designed to display the received data online and store them for further use. The second part of the ground station, the command and control console (CCC), is used to pilot the IoT carrier and remotely control the gimballed camera. In our system, WMMS and CCC are separate devices that are connected to the air station through separate networks: the production network, described in the next section, and the control network, respectively. As the WMMS, a laptop computer was used. As the CCC, a Turnigy 5X was used, which offers five-channel connectivity using a network based on secure Frequency-Hopping Spread Spectrum (FHSS), operating in the 2.4 GHz band. The second endpoint of the control network is the remote control receiver, which is shown in Figure 1 hanging on the left side of the UAV.

Improving the transmission quality usually involves the use of quality of service (QoS) mechanisms. In a flying monitoring system, the QoS assurance can be used at the application layer for both sensor data transmission and video transmission. In this research, MQTT QoS level 0 was used for sensor data transmission because the reliable transport protocol was able to provide QoS. However, higher levels of MQTT QoS are enabled in our system and can be used as needed. In the case of video, adaptive coding was used, in which we defined our own rules. At lower layers, there is no point in defining QoS rules on a node because WebRTC sends one aggregated stream. If necessary, the sensor data stream and video stream can be separated, allowing the WebRTC streams to take advantage of the Differentiated Services (DiffSev) mechanism at the network layer, supported by QoS in the 802.11 network. An analysis of similar cooperation is presented in [9].

### 2.2. Experiments

Experiments were carried out in the parking lot of the AGH University of Krakow, Poland to assess whether there are any benefits and it makes sense to use external transceivers to increase the range and quality of air-ground transmission. During the experiments, the air station transmitted IoT data and 4K video to the ground via the production network.

**Figure 2:**
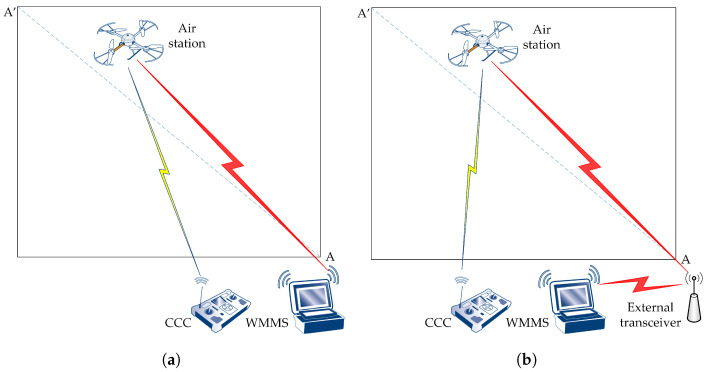
Testbed: (**a**) scenario S1; (**b**) scenario S2.

Test flights took place over a square parking lot measuring approximately 70 by 70 m, located on the campus of AGH University. The two corners of the square, located diagonally, were highlighted (Figure 2). Point A was the beginning of the test flight route, and point A’ was its end. The length of the entire route A-A’ was 100 m.

The experiments were carried out according to two scenarios, differing in the presence of an external transceiver and, consequently, in the architecture of the 802.11ac network used as the production network. These are as follows:

- scenario S1: transmissions are carried out without an external transceiver,
- scenario S2: transmissions are carried out using an external transceiver.

In scenario S1, the WMMS was placed at point A and the production network was built as an independent BSS (IBSS) (Figure 2a). In scenario S2, the WMMS was located approximately one meter away from point A, while the external transceiver was located at point A. Due to the presence of the external transceiver, the production network in scenario S2 consisted of two BSSs, and the external transceiver relayed the packets between the air station and the WMMS (Figure 2b).

The network interfaces at the WMMS and at the air station were the same in both scenarios. The WMMS used the IEEE 802.11 Intel^®^ Dual Band Wireless-AC 7260 network interface. The air station used the IEEE 802.11ac dual band (2.4 GHz and 5 GHz) network interface built into the SBC. The network interface at the air station is able to support beamforming, so the external transceiver could use explicit beamforming, managing signal power better than the implicit one. As the external transceiver, a NETGEAR Nighthawk X4 R7500 AC2350 dual-band access point was used, which is equipped with four high-performance external antennas and performs both explicit and implicit beamforming.

During the experiments, the air station moved along the straight 100 m long A-A’ trajectory at an altitude of 15 m ± 10 cm (measured with a barometric altimeter), sending air-to-ground the aggregated stream of IoT data and 4K video. In each flight, the amount of transmitted data and spatio-temporal metadata were collected. On this basis, the total throughput of the aggregated stream received by the WebRTC-based monitoring application running on the ground station, expressed in megabits per second (Mbps), was calculated. Additionally, the received signal strength indicator (RSSI) value expressed in decibel-milliwatts (dBm) and the available data rate expressed in Mbps were read every half a second at the air station. These values were collected along with their spatio-temporal metadata.

The experiments were repeated at least a week apart, on the same day of the week, at different times of the day. Choosing the same day of the week allowed for a clear and simple way to ensure the randomness of weather conditions. Several dozen flights were made during the tests, with flights on the same day giving similar results. Flights from five flying days were selected for further analysis, during which air-to-ground transmissions were carried out in various circumstances: at different times of the day (morning flights and afternoon flights), in various weather conditions: temperature from 5 to 26 degrees Celsius, relative air humidity from 45% to 82%, with various cloud cover: sunny, partly cloudy, cloudy. To enable throughput measurement in stable conditions and to facilitate comparison of results from different flights, measurement points were designated on the A-A’ route where the air station hovered for two seconds.

Measuring points were established every five meters. The first one was at point A, the last one was at point A’. The results presented in the following sections are based on RSSI and available data rate readings, as well as total throughput measurements, which were collected at the measuring points on each of the five selected flying days, one S1 scenario transmission and one S2 scenario transmission for each day. During each flight, both MQTT messages carrying data from sensors (two bursts of 27 messages per second, i.e., 54 messages per second) and RTP packets carrying video frames were transmitted. The number of RTP packets depended on the current state of the air-ground channel and was in the best case 2180 packets per second (scenarios S1 and S2), and in the worst case 980 packets per second (scenario S1) and 1575 packets per second (scenario S2).

RSSI and available data rate were collected at a given measurement point over five different flying days and then averaged for that point. The total throughput was calculated for each two-second hover of the air station at a given measuring point and averaged over five flights. The total throughput of the aggregated stream, expressed in megabits per second (Mbps) is the sum of the Internet Protocol (IP) packet sizes received at the ground station while the air station was hovering over the measurement point, divided by the time of transmission of these packets. Because the stream has been aggregated, there is no distinction at the IP level between sensor data transmission and video transmission.

### 2.3. Measures of Improvement

Let the index S1 be the measurement made during the experiment carried out according to scenario S1, i.e., without an external transceiver, and the index S2 be the measurement made during the experiment carried out according to scenario S2, i.e., with the use of an external transceiver. As mentioned in the previous section, the S1 scenario experiment and the S2 scenario experiment were performed under the same circumstances, with the same or nearly the same network conditions, and without the change in the network interfaces at the air station and the WMMS.

Let $d_{h}$ be the horizontal distance between the air station and point A and $d_{k}$ be the horizontal distance between the *k*-th measurement point and point A. If the air station is hovering over the *k*-th measurement point, the horizontal distance $d_{h}$ between the air station and point A is (in meters):

(1) $$d_{h}=d_{k}=\left(k-1\right)\cdot 5\;\left[m\right],\;k=1,\;2,\;\ldots ,\;21.$$

As a measure of the RSSI improvement resulting from the use of an external transceiver, the increment ΔRSSI will be taken, defined as the difference between the average RSSI value obtained for the experiment carried out according to the S2 scenario and the average RSSI value obtained for the experiment carried out according to the S1 scenario when the air station hovered over the *k*-th measuring point:

(2) $$\Delta RSSI\left(d_{k}\right)=RSSI_{S2}\left(d_{k}\right)-RSSI_{S1}\left(d_{k}\right).$$

Similarly, as a measure of the improvement in the available data rate resulting from the use of an external transceiver, the difference ΔADR will be taken:

(3) $$\Delta ADR\left(d_{k}\right)=ADR_{S2}\left(d_{k}\right)-ADR_{S1}\left(d_{k}\right),$$

where $ADR_{S1}\left(d_{k}\right)$ is the average available data rate value obtained for the S1 scenario experiment when the air station hovered over the *k*-th measuring point and $ADR_{S2}\left(d_{k}\right)$ is the average available data rate value obtained for the S2 scenario experiment when the air station hovered over the *k*-th measuring point.

The measure of the improvement in the total throughput of the air-to-ground transmission as a result of the use of an external transceiver will be the difference ΔThr defined as follows:

(4) $$\Delta Thr\left(d_{k}\right)=Thr_{S2}\left(d_{k}\right)-Thr_{S1}\left(d_{k}\right),$$

where $Thr_{S1}\left(d_{k}\right)$ is the average total throughput value obtained for the S1 scenario experiment when the air station hovered over the *k*-th measuring point and $Thr_{S2}\left(d_{k}\right)$ is the average total throughput value obtained for the S2 scenario experiment when the air station hovered over the *k*-th measuring point.

The analysis will also use the relative measures δRSSI, δADR and δThr, which express the values of ΔRSSI, ΔADR and ΔThr as a percentage of the corresponding values measured during the experiments conducted according to the S1 scenario under the same circumstances, when the air station hovered over the *k*-th measuring point, i.e., at the same horizontal distance $d_{h}$ between the air station and point A. Relative measures of improvement are determined only for the values of $RSSI(d_{k})$, $ADR(d_{k})$ and $Thr(d_{k})$ different from zero.

The relative improvement of the RSSI, denoted as δRSSI, is ΔRSSI related to the average RSSI value obtained for the experiments carried out according to the S1 scenario when the air station hovered over the *k*-th measurement point:

(5) $$\delta RSSI\left(d_{k}\right)=\frac{RSSI_{S2}\left(d_{k}\right)-RSSI_{S1}\left(d_{k}\right)}{\left|RSSI_{S1}\left(d_{k}\right)\right|}\cdot 100\%,$$

Similarly, the relative improvement in the available bit rate δADR is ΔADR related to the average available bit rate value measured during the experiments conducted according to the S1 scenario, when the air station hovered over the *k*-th measuring point:

(6) $$\delta ADR\left(d_{k}\right)=\frac{ADR_{S2}\left(d_{k}\right)-ADR_{S1}\left(d_{k}\right)}{ADR_{S1}\left(d_{k}\right)}\cdot 100\%,$$

Finally, the relative improvement of the total throughput δThr is equal to ΔThr related to the average value of the total throughput measured during the experiments conducted according to the S1 scenario when the air station is hovering over the kth measurement point:

(7) $$\delta Thr\left(d_{k}\right)=\frac{Thr_{S2}\left(d_{k}\right)-Thr_{S1}\left(d_{k}\right)}{Thr_{S1}\left(d_{k}\right)}\cdot 100\%,$$

The above measures of improvement, both absolute and relative, take a positive value when the use of an external transceiver resulted in an improvement in the parameter (RSSI, available bit rate, total throughput) and a negative value when the use of an external transceiver resulted in a deterioration of the parameter. If the use of an external transceiver did not change the parameter, the corresponding measure of improvement is zero.

## 3. Results

This section compares transmissions carried out without (scenario S1) and with (scenario S2) an external transceiver. The comparison was performed in terms of the RSSI, the available data rate, and the throughput of aggregated data consisting of bulk data from the UAV camera (20 Mbps) and sensor data (100 kbps in total). Measurements were collected at the network interface level, operating system level, and application level, respectively. The values are presented as a function of the horizontal distance $d_{h}$ between the air station and the starting point of the flight route (point A).

### 3.1. Relative Signal Strength

**Figure 3:**
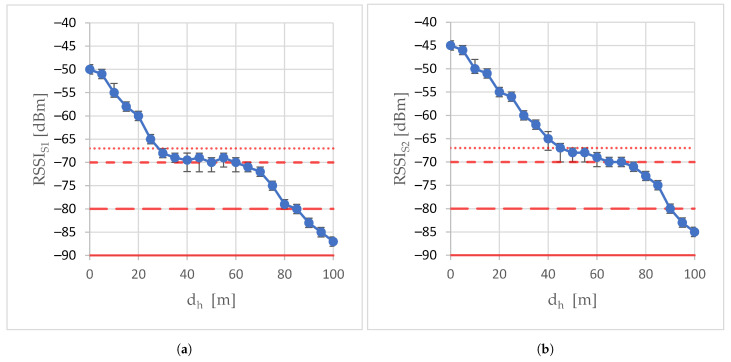
Relative signal strength (RSSI) in scenarios: (**a**) scenario S1; (**b**) scenario S2.

Figure 3 shows the RSSI readings taken at the air station as the air station moved away from point A towards point A’. Because the signal strength decreases with the distance between the air station and the ground one, the graph of the RSSI as a function of the horizontal distance $d_{h}$ between the air station and point A is a monotonically falling curve. In both the S1 scenario experiment (Figure 3a), when the external transceiver was not used, and in the S2 scenario experiment, when it was used (Figure 3b), RSSI readings passed three consecutive limits. They were as follows: the −67 dBm lossless real-time media streaming limit (dot line in Figure 3), the −70 dBm lossless non-real-time data transmission limit (dash line), and the limit of −80 dBm for basic connectivity (long dash line). During experiments, the RSSI never exceeded −90 dBm of the limit of connectivity (solid line in Figure 3).

When the two first limits were exceeded, the transceiver built into the WMMS (scenario S1) and the external one (scenario S2) took intensive countermeasures to try to stop further signal degradation, which was effective for some distances. The slopes of the middle sections of the RSSI characteristics are so much smaller than the slopes of the other two sections. As a result, this section of the RSSI curve can be considered quasi-constant.

**Figure 4:**
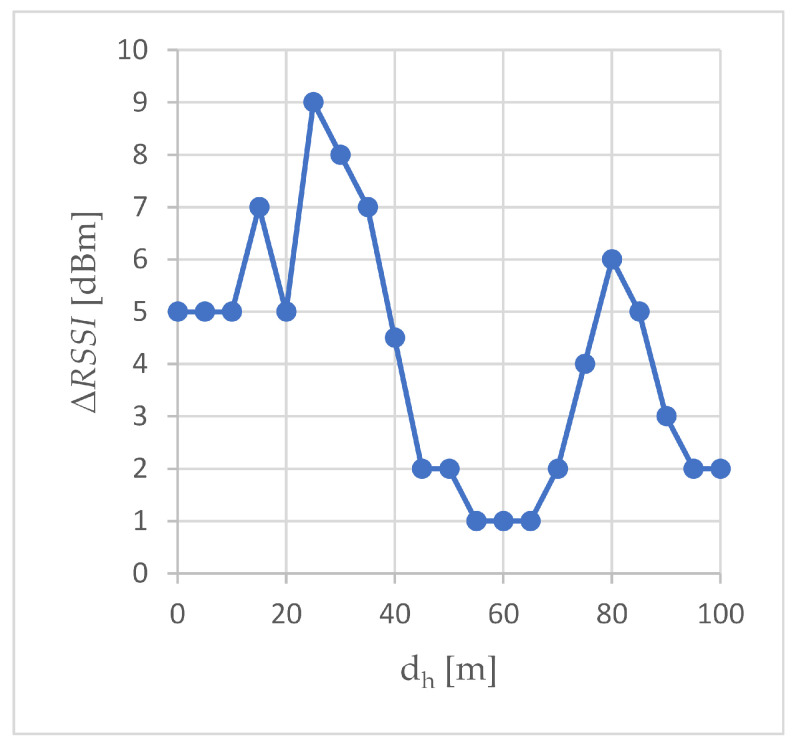
The improvement in the measured RSSI value.

The ΔRSSI plot (Figure 4) shows the improvement in RSSI caused by the use of the external transceiver when the air station was moving along the A-A’ trajectory. In all experiments, the effect of using an external transceiver was always positive (ΔRSSI was always greater than zero), meaning that the use of an external transceiver always resulted in improvement in the RSSI. The degree of this improvement was different for different distances between the air station and the ground one and generally depended on differences in RSSI characteristics collected at the air station when the WMMS was operating with and without the external transceiver. The most important differences affecting ΔRSSI are different initial RSSI values (measured when the air station was just above the ground one), different slopes of the relevant sections of the RSSI curves, and differences in the ability of each transceiver to counteract signal degradation and, as a result, to keep the RSSI at the air station close to the −70 dBm limit despite the growth in $d_{h}$. As an effect, when the air station followed trajectory A-A’, both increases and decreases in the ΔRSSI were observed, and the constant or quasi-constant sections of the ΔRSSI curve, as well.

The same rate of descent of the RSSI curves over a given section of the route resulted in a constant ΔRSSI. This can be seen, for example, at the very beginning of the A-A’ trajectory (the first 10 m, ΔRSSI of 5 dBm). The ΔRSSI minimum (ΔRSSI of 1 dBm), which took place between the 55th and 65th meters of the A-A’ trajectory, occurred when both the RSSI in the S1 scenario and the RSSI in the S2 scenario were on quasi-constant sections of their curves.

The increase in the ΔRSSI occurred when the RSSI curve in scenario S1 fell much faster than the RSSI curve in scenario S2. Such an increase was the cause of two maxima, global and local, on the ΔRSSI curve shown in Figure 4. The global maximum (9 dBm) occurred at $d_{h}$ equal to 25 m. In this case, the growth of the ΔRSSI ended when the RSSI reading in the S1 scenario passed the −67 dBm limit ($d_{h}$ equaled 30 m), as a result of which the RSSI entered a quasi-constant section of the RSSI characteristic. Since in scenario S2 the RSSI was still above the −67 dBm limit, on the descending section of the RSSI characteristics, a reduction in the ΔRSSI occurred (up to 2 dBm for $d_{h}$ of 45 m). The other maximum, i.e., the local one, was caused by a sharp drop in the RSSI in scenario S1, while the RSSI in scenario S2 was still at the quasi-constant section of the curve. The ΔRSSI increases up to 6 dBm at $d_{h}$ = 80 m, and then decreases when the RSSI in the S2 scenario fell well below the −70 dBm limit. The next, −80 dBm of the limit of basic connectivity (long dash line in Figure 3) was achieved at 85 m from point A in scenario S1, and at 90 m from this point in scenario S2. After exceeding this limit in scenario S2, the ΔRSSI decreased to 2 dBm and remained at this level when the air station reached point A’.

### 3.2. Available Data Rate

**Figure 5:**
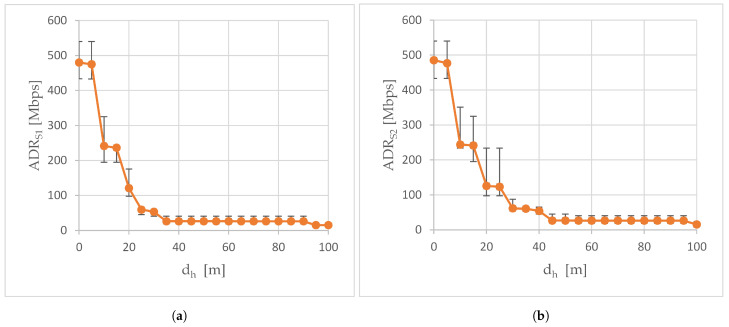
Available data rate read in scenarios: (**a**) scenario S1; (**b**) scenario S2.

Figure 5 shows the mean available data rate calculated from the values of the available data rate read from the controller of the network card of the flying IoT system when the air station flies away from the ground station along the line A-A’, where A was the location of the ground station. Both in the S1 scenario (Figure 5a), when the external transceiver was not used in air-to-ground transmissions, and in the S2 experiment scenario, when it was used (Figure 5b), as the horizontal distance of the air station from point A increases, the curves of available data rate descend exponentially and then stabilize at around 26 Mbps to drop to around 15 Mbps at the end of trajectory A-A’.

**Figure 6:**
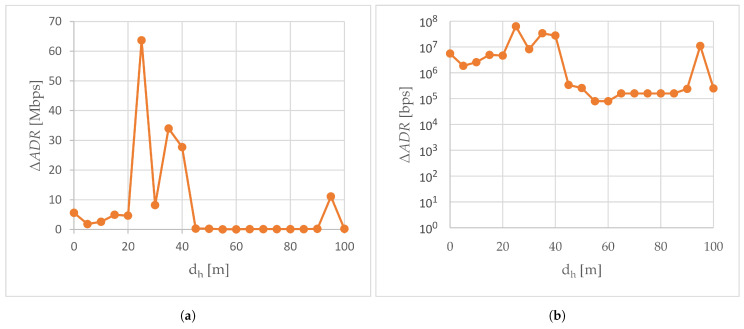
The improvement in the available data rate. ΔADR is plotted: (**a**) on a linear scale; (**b**) on a logarithmic scale.

The ΔADR presented in Figure 6 is the improvement in the available data rate caused by the use of an external transceiver. In the case of trajectory A-A’, the values of ΔADR were always greater than zero, which means that the support of the WMMS’s built-in transceiver with the external transceiver has increased the available data rate (here 80 kbps to nearly 64 Mbps). Positive values of the ΔADR were achieved due to the longer persistence of a slower rate of decline of the exponential section of the available data rate curve, longer persistence of the exponential decline (i.e., delay in transition to a quasi-constant section of the curve), and longer remaining at 26 Mbps in the case of the use of the external transceiver.

Since the slopes of the curves for both scenarios were roughly the same for the first 20 m of the A-A’ trajectory, on this section of the A-A’ route the use of an external transceiver resulted in only a slight improvement in the available data rate (ΔADR from 1.9 to 5.6 Mbps). After exceeding 20 m of the $d_{h}$ distance, the rate of decline of the exponential curve in the S1 scenario increased, which caused a large increase in ΔADR from 4.7 Mbps ($d_{h}$ of 20 m) to the global maximum of the ΔADR curve depicted in Figure 6, i.e., 64 Mbps ($d_{h}$ of 25 m). As a reminder, in the same horizontal distance $d_{h}$ = 25 m, the largest increase in ΔRSSI was recorded (Figure 4). The local maximum of the ΔADR curve (30 Mbps), which occurred at the measurement point located 35 m from point A, corresponded to the large ΔRSSI (7 dBm) recorded at this measurement point. However, since RSSI is not the only parameter considered when determining the available data rate, the shapes of the ΔRSSI and ΔADR curves are different.

As the horizontal distance of the air station from the A point continued to increase, starting from $d_{h}$ equal to 35 m in the S1 scenario and $d_{h}$ equal to 45 m in the S2 scenario, the average available data rate was only around 26 Mbps. In both cases, the decrease in the available data rate was associated with the RSSI exceeding the −67 dBm limit of lossless real-time media streaming.

The available data rate remained at 26 Mbps for almost the rest of the A-A’ route. Only at the end of the A-A’ route (for $d_{h}$ of 95 m in the S1 scenario and $d_{h}$ of 100 m in the S2 scenario, i.e., in the place where the RSSI reached −85 dBm), did it drop to 15 Mbps. As a result, from $d_{h}$ equal to 45 m, the value of ΔADR ranged from 80 kbps to 340 kbps. Only at the penultimate measurement point, for $d_{h}$ equal to 95 m, did a small ΔADR increment (the second local minimum of the above 11 Mbps) occur, related to a longer stay on a quasi-constant section of the available bit rate curve in the S2 scenario.

### 3.3. Total Throughput of WebRTC Transmissions

During experiments, the aggregated stream that consisted of a 4K video stream and the non-media flow that includes data from sensors and their metadata was transmitted air-to-ground through the production network. The total bit rate of the aggregated stream was set to 20 Mbps and, due to the congestion control implemented by the WebRTC video service, it may have been reduced on-the-fly.

**Figure 7:**
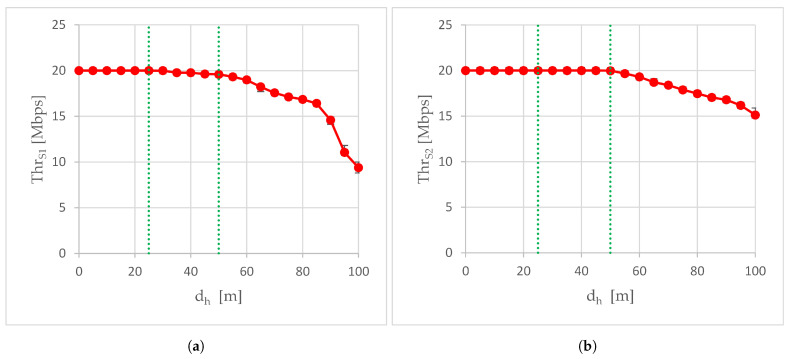
Total throughput calculated in scenarios: (**a**) scenario S1; (**b**) scenario S2. Green dotted lines mark the boundaries of the areas defined in Section 4.

Figure 7 presents the total throughput of the aggregated stream as a function of the horizontal distance $d_{h}$ between the air station and point A, when the air station moves away from point A along the A-A’ route. Since the aggregated traffic was rate-limited, although the available data rates were set from about 26 Mbps to about 480 Mbps on almost the entire A-A’ route, the total throughput of the aggregated stream was at most 20 Mbps. At the beginning of the A-A’ trajectory, where the operating system set high available data rates, the air-to-ground transmission was error-free, which resulted in maximum throughput. In the absence of the external transceiver, the total throughput remained at 20 Mbps for the first 25 m of the A-A’ trajectory (S1 scenario, Figure 7a), while the use of the external transceiver doubled the length of the A-A’ trajectory segment, where the total throughput was 20 Mbps (Scenario S2, Figure 7b). Starting from the 30th meter of the A-A’ trajectory in the S1 scenario and the 55th meter of this trajectory in the S2 scenario, the throughput curve began to descend due to packet loss and the response of the congestion control to packet loss. This was correlated with the RSSI exceeding the −67 dBm limit of lossless real-time media streaming (Figure 3). The throughput curve did not fall evenly, but its slope changed on subsequent sections of the A-A’ route.

**Figure 8:**
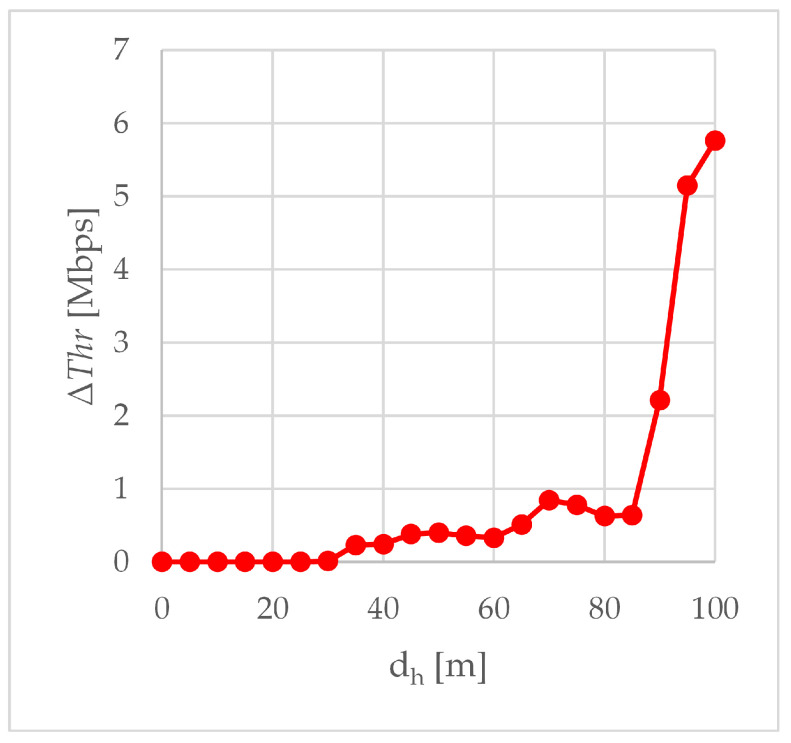
The improvement in total throughput ΔThr of air-to-ground transmissions.

Figure 8 shows the improvement in the total throughput ΔThr when the transceiver built into the WMMS was supported with the external transceiver. Since for the first 25 m of trajectory A-A’ the total throughput was 20 Mbps in both scenario S1 and scenario S2, on this section of the route A-A’ the use of an external transceiver did not result in a throughput improvement (ΔThr equals zero up to $d_{h}$ of 25 m). Starting from the 30th meter of the A-A’ trajectory, ΔThr was always greater than 0, and it increased and decreased with the increase in the $d_{h}$, due to differences in the slope of the throughput curves when WMMS was operated with and without an external transceiver.

The first, relatively small increase in total throughput was related to the slow decline of the throughput curve in the S1 scenario while maintaining a throughput of 20 Mbps in the S2 scenario. As a result, ΔThr grew up to 400 kbps for $d_{h}$ equal to 50 m. At the 55th meter of the A-A’ trajectory in the S2 scenario, the throughput started to decrease, which resulted in a decrease in ΔThr to 329 kbps at the 60th meter of the A-A’ route. The next sequence of rise and fall of ΔThr occurred between the 60th and 85th meters of trajectory A-A’, with a local maximum at 70 m (ΔThr of 843 kbps). After exceeding 85 m of the A-A’ trajectory, in the S1 scenario, the relatively gentle slope of the throughput curve began to fall steeply, resulting in an increase in ΔThr to 5.76 Mbps at point A’ ($d_{h}$ equal to 100 m). This is expected to be the beginning of another rise and fall sequence of ΔThr. It is worth noting here that at the 85th meter of the A-A’ trajectory, in the S1 scenario, the RSSI curve was just at the −80 dBm limit of basic connectivity. However, although in the S2 scenario, the RSSI curve reached this limit at the 90th meter of route A-A’, this did not cause such a strong change in the slope of the throughput curve.

## 4. Discussion

In the previous section, the results of the improvement achieved by the use of an external transceiver were analyzed in terms of RSSI, available data rate and throughput of aggregate media stream and non-media flow. The analysis was carried out at a distance from 0 to 100 m, with a step of 5 m. The choice of 100 m as the maximum horizontal distance between the air station and the ground one resulted directly from preliminary tests carried out at distances of 0–250 m. It was shown that the maximum improvement of the total throughput occurs near 100 m. Since, in addition, the Thr values obtained for 100 m in the S1 scenario were assessed as still useful for SAR missions, 100 m was selected as the final distance.

**Figure 9:**
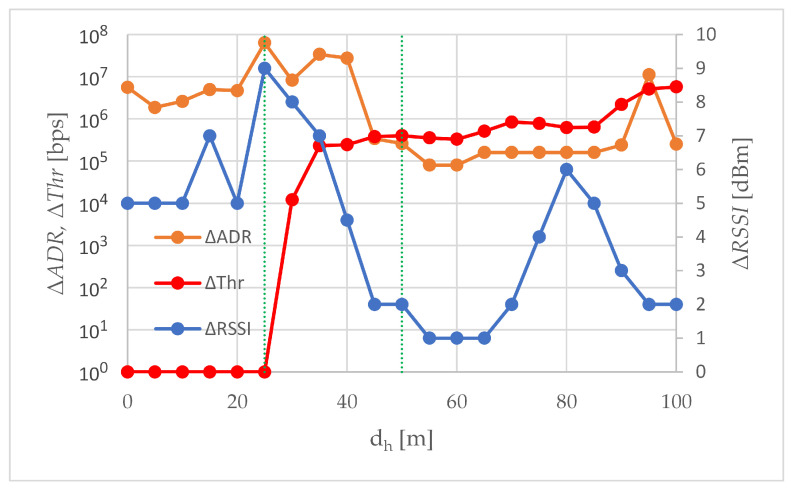
The absolute increment achieved by use of an external transceiver. Green dotted lines mark the boundaries of the areas.

The absolute improvements ΔRSSI, ΔADR and ΔThr are compared in Figure 9. The available data rate increment (ΔADR) and throughput increment (ΔThr) are expressed in b/s and presented on a logarithmic scale. The RSSI increment (ΔRSSI) is shown on a linear scale. Although all three charts compared in Figure 9 are different in nature, three areas can be distinguished in the drawing. Two of them correspond to small and large distances from point A’, and therefore, to small and large error rates, and the third one is the transition area between these two.

The first area covers the beginning of the trajectory A-A’, a short distance from point A, up to 25 m. This is an area of error-free transmission, both with and without the use of an external transceiver. As an effect, in both scenarios, the total throughput of the WebRTC transmission is the same, so when the horizontal distance from point A is up to 25 m, there is no throughput improvement and ΔThr is zero. The lack of transmission errors causes the available data rate to be determined solely on the basis of RSSI, which is visible in Figure 9: the shape of the ΔADR curve (increase in the available rate) roughly resembles the shape of the ΔRSSI curve (increase in RSSI), especially between 10 and 25 m of the A-A’ route. In the first area, there were the largest observed absolute values of RSSI improvement, up to 9 dBm. The maximum absolute improvement in RSSI was found at the edge of the first area, 25 m from point A.

Summarizing, in the first area, if the production network is well-dimensioned, the greatest RSSI improvement resulting from using an external transceiver will not be exploited by a single air-to-ground WebRTC transmission. The rate-limited nature of the aggregated WebRTC traffic has meant that despite good performance in ΔRSSI and ΔADR, there is no need for using an external transceiver in the first area if it were to transmit traffic only from that one air station. However, the reserves in RSSI and the available data rate offered by the external transceiver may be useful if the air station also acts as an intermediate node in transmitting external traffic, in addition to its own traffic coming from its equipment. An air station flying in the first area could then perform its normal monitoring tasks and potentially be a flying access point or flying intermediate node relaying incoming traffic from other air stations to the ground station.

As the air station moved away from point A, the station passed first through a transition area and then through a second area. In the transition area, after exceeding 25 m from point A, transmission errors begin to appear in the S1 scenario, increasing with the distance from point A. The transmission in the S2 scenario in the transition area remains error-free. The occurrence of transmission errors in scenario S2 observed at a measurement point 55 m away from point A, signals the completion of the transition from the first area to the second one. It should be noted that while the use of an external transceiver allowed for an increase in the horizontal distance, at which the RSSI real-time lossless multimedia streaming limit of −67 dBm was exceeded, from 25 m to 45 m (Table 4), i.e., by 80%, the error-free transmission limit increased from 25 m to 50 m (Section 3.3), i.e., by 100%. The ΔRSSI peak visible in Figure 9 for distances from 60 to 100 m is not due to the sudden improvement of RSSI in scenario S2, but to the collapse of the RSSI characteristics in scenario S1. Between 70 and 80 m, the characteristics in S1 decreased by 7 dBm. A similar breakdown, but in the S2 scenario, occurs after exceeding 80 m, where between 80 and 90 m the RSSI dropped by 4 dBm. This caused the ΔRSSI to return to 2 dBm, where it persisted up to 100 m.

The clear drop in ΔRSSI in the transition area shown in Figure 9 causes a drop in ΔADR only at the first measurement point in this area ($d_{h}$ equal to 30 m). Neither the further decrease in ΔRSSI in the transition area nor the subsequent increase in ΔRSSI to 6 dBm and then decrease to 2 dBm observed in the second area were visualized in the shape of the ΔADR curve. This is because, from the very beginning of the transition area, the available data rate in the S1 scenario was influenced by both PER and RSSI. As the distance of the air station from point A increases, the packet error rate becomes increasingly important in ADR calculations. Therefore, although at the beginning of the A-A’ trajectory ΔADR kept up with the ΔRSSI, with subsequent meters separating the air station from point A, the shape of ΔADR begins to diverge from the shape of ΔRSSI. In the final phase of the flight, at high $d_{h}$, the shape of ΔADR curve is closer to the shape of ΔThr curve.

Because the available data rate values are set for a given RSSI and a given error rate, and they are tabulated values, the greatest improvement in available data rate results from shifting the distance at which the newly available data rate value is set. In the transition area and in the second area, in the case of the S2 scenario, this occurs over longer distances than in the case of the S1 scenario. As seen in Table 5, this shift is approximately 10 m in the transition area and approximately 5 m in the second area. At a given available bit rate, the use of an external transceiver resulted in a relative improvement in the transmission range of 33 to 40 percent in the transition area and greater than 5.5 percent in the second area. To simplify the assessment of range improvement, Table 5 includes the most frequently repeated value (modal value, or mode) among all available data rate readings taken at a given distance $d_{h}$, rather than the arithmetic mean.

Starting from the 30th meter of the A-A’ route, the use of the external transceiver increases the total throughput of WebRTC transmissions. At the 30th meter, it is an increase of ten kbps, from the 35th meter inclusive, an increase of hundreds of kbps, and in the last 10 m of the A-A’ trajectory, ΔThr reaches several Mbps (Figure 9). The smallest absolute improvement in total throughput results from very small PER values. Since the throughput reduction is only 0.5 per mille of the target bit rate, in many applications such a reduction can be considered negligible, and then the measurement point $d_{h}$ = 30 m can be considered as the approximate boundary of the first area. The greatest absolute improvement in total throughput was obtained at point A’, where ΔThr was 5.76 Mbps. In the case of the S2 scenario, the useful transmission range, allowing maintaining the quality of service and ensuring seamless transmission of data from the UAV (4k video image with a high frame rate and data from sensors), was maintained up to $d_{h}$ of 100 m, while in the case of the S1 scenario, after exceeding 85 m of horizontal distance from point A, throughput began to decline rapidly. Concluding, the external transceiver fulfilled its purpose of increasing the range and quality of WebRTC air-to-ground transmissions. Concluding, the external transceiver fulfilled its purpose of increasing the range and quality of WebRTC air-to-ground transmissions. This conclusion was confirmed by the results of the Wilcoxon rank sum test conducted using MATLAB R2022a (The MathWorks, Inc., Natick, MA, USA) software. The test rejected the null hypothesis of equality of medians at the 5% significance level, and the low *p*-value of 0.0002 does not provide grounds to question the rejection of the hypothesis. The effect of improvement in total throughput, observed after using the external transceiver is, therefore, statistically significant.

**Figure 10:**
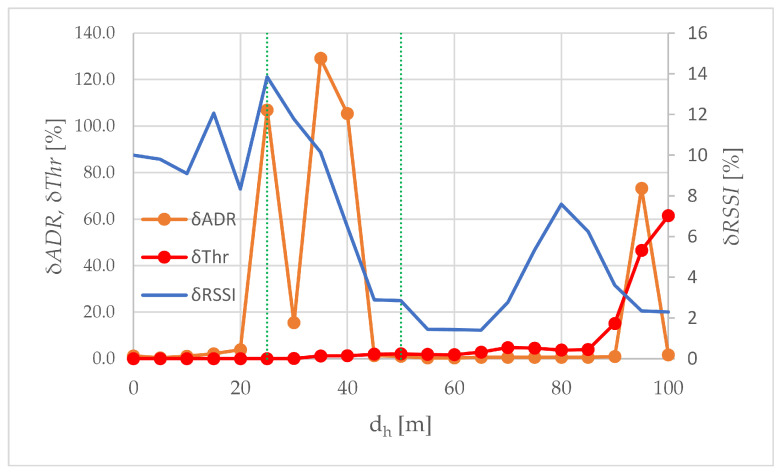
The relative improvement achieved by use of an external transceiver. Green dotted lines mark the boundaries of the areas.

Figure 10 compares the relative improvement (δRSSI, δADR, and δThr) as defined by Formulas (5), (6) and (7), respectively. All values are expressed as a percentage of the value measured when the air-ground transmission does not use an external transmitting and receiving device. As can be seen in Figure 10, the greatest relative improvement in RSSI occurred in the first area, i.e., on the section between point A and the 25th meter of route A-A’. This corresponds to the large absolute improvements in RSSI, from 5 to 9 dBm, shown in Figure 9. Although the large absolute improvement in the available data rate also occurred up to the 20th meter of route A-A’ (Figure 9), is not reflected in Figure 10. The use of an external transceiver in the first 20 m of the A-A’ only a little increased the already very high available data rate. At these distances, the external transceiver can provide the reserve of available bit rate for unexpected events.

The large relative improvement in available data rate occurs mainly in the transition region and at the end of the A-A’ trajectory. This is because the largest ΔADR is caused by the shift in the horizontal distance from point A, at which the new value of the available data rate is set (Table 5), and the differences between the available data rate averaged over the performed experiments are much smaller. A significant relative improvement in throughput is visible at the end of the A-A’ trajectory. This improvement is an effect of the collapse of throughput characteristics in scenario S1, visible in Figure 7a after exceeding 85 m when RSSI drops below the limit of −80 dBm for basic connectivity (Figure 3a). The greatest relative improvement in total throughput that was achieved at point A’, where ΔThr was 5.76 Mbps, has resulted in δThr as high as 61.46%. The RSSI in scenario S1 was then −87 dBm.

External transceiver was the intermediate node located close to the WMMS that emulates the WMMS’s transmitter. The improvement in RSSI, ADR and total throughput is similar to what would be achieved by replacing the network interface at the WMMS with an interface from the external transceiver, minus the cost of communication between the WMMS and the external transceiver. Comparing the obtained relative improvement in the total throughput of line-of-sight air-to-ground transmissions with that known from the literature, it can be seen that the experiments achieved better results than the relative improvement in throughput (gain) obtained during experiments with line-of-sight air-to-ground transmissions with and without beamforming, reported in [41]. In [41] such large gains were reported only for non-line-of-sight transmissions. This proves that the cost of communication between the WMMS and the external transceiver can be considered negligible.

We have configured the access point acting as an external transceiver to the range extender mode. There was a clear difference in range and quality of transmission in favor of the external transceiver because the cost of communication between the laptop and the range extender was too high. The first assumption, “improving the transmission range and quality must be based on a factor mentioned in [31] other than propagation”, was met thanks to beamforming, but the second assumption, “such intermediate nodes must be able to transmit and receive simultaneously ”, has not been fulfilled. Although RSSI was improved, it did not translate into performance in higher network layers.

**Figure 11:**
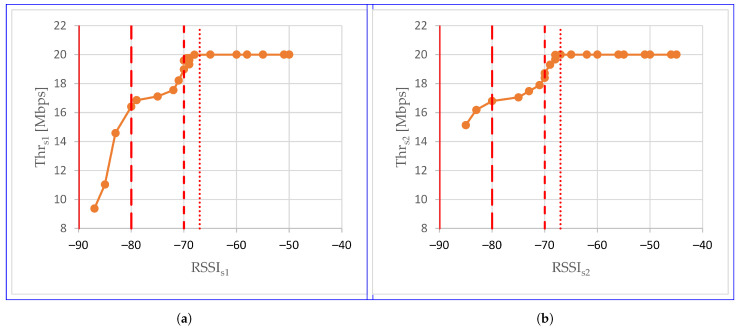
Total throughput as a function of RSSI: (**a**) scenario S1; (**b**) scenario S2.

Figure 11 combines average total throughput and average RSSI obtained for the same measurement point. While the graph points correspond to the RSSI and total throughput values obtained at a given measurement point, the graph lines connect adjacent measurement points. Moving along the graph lines corresponds to moving along the straight line A-A’. The extreme points, which have only one neighboring point, correspond to points A’ (leftmost point) and A (rightmost point). The vertical lines visible in the graph indicate subsequent limits, similar to the horizontal lines in Figure 3. And so: the dot line indicates the −67 dBm lossless real-time media streaming limit, the dashed line indicates the −70 dBm lossless non-real-time data transmission limit, the long dash line indicates the −80 dBm basic connectivity limit, the solid line indicates the −90 dBm connectivity limit.

As shown in Figure 11, for the same RSSI, the total throughput of the aggregated stream may be different. Differences may appear between scenarios. This is most visible on the left side of the graph, i.e., near point A’, where the Thr in the S2 scenario significantly exceeds the Thr in the S1 scenario. For the RSSI of −85 dBm, these are 11 Mbps (S1) and 15.1 Mbps (S2). The further the air station was from point A’, the smaller the differences in total throughput were. For RSSI of −83 dBm it was 14.6 Mbps (S1) and 16.2 Mbps (S2), and for RSSI of −80 dBm it was 16.4 Mbps (S1) and 16.8 Mbps (S2). For RSSI of −75 dBm, the difference in total throughput decreased to zero. In both scenarios, the Thr was 17.1 Mbps. The trend then reversed: Thr in scenario S1 exceeded Thr in scenario S2 and the differences in the total throughputs achieved in scenarios S1 and S2 started to increase. For RSSI of −70 dBm, the Thr was 19 Mbps (S1) and 18.4 Mbps (S2).

An RSSI of −70 dBm is an example of the fact that differences in the total throughput values obtained for the same RSSI value not only occur between S1 and S2 scenarios but can also occur within S1 and S2 scenarios. An RSSI of −70 dBm occurred twice in each scenario: in the S1 scenario at two measurement points 10 m apart and in S2 at adjacent measurement points, i.e., at a distance of 5 m. In both cases, the total throughput was higher at the measurement points located closer to point A: 1.96 Mbps (S1) and 1.87 Mbps (S2). For an RSSI of −68 dBm, even if the RSSI increased, the same total throughput of 20 Mbps was still obtained. This is due to the rate-limited nature of the aggregated stream. Without further research, it is difficult to clearly indicate the reasons for the difference in total throughput at the same RSSI value. Perhaps RSSI is not the only contributing factor. Or maybe it is due to the nature of RSSI. RSSI, as the name suggests, is an index, an integer. The lack of a fractional part can lead to a situation where one RSSI value can represent two signal strengths that differ by almost 1 dBm.

## 5. Conclusions

The aim of this paper was to demonstrate that, under certain conditions, the use of an intermediate node can improve the range and quality of transmission between the UAV and the ground station, observed at the application layer level, even if the intermediate node is located too close to the end node to obtain the benefits of splitting the air-ground link into segments. Its novelty in relation to previous works consists in the use of an intermediate node in a non-standard position that is considered incorrect by common sense.

The concept of the external transceiver proposed in this paper consists in extending the range and improving the quality of direct air-to-ground transmission in the IEEE 802.11 standard by using an intermediary device with better transmission parameters than the network interface originally built in the ground station. While classic solutions such as range extenders and nodes of mesh or chain networks are intended to shorten the air-to-ground distance, the external transceiver is close enough to the ground station interface to be considered a part of a ground station, and the original length of the air-to-ground segment is roughly preserved. This makes it possible to use an external transceiver to increase the range and quality of transmission in situations where the use of classic ground network nodes would be difficult or impossible, e.g., in a flooded or fire area.

In this paper, we have analyzed the air-to-ground transmission of aggregated congestion- controlled traffic consisting of 20 Mbps video traffic and 100 kbps sensor data traffic. Aggregated traffic was transmitted from an air station to a ground station of the flying monitoring system over an IEEE 802.11ac network. The transmission was carried out using WebRTC technology, i.e., WebRTC’s media logical channel for video transmission and WebRTC’s web logical channel for transmission of data coming from sensors, and evaluated in field experiments, during which an access point enabling beamforming was employed as an external transceiver. Comparative transmissions were carried out without an external transceiver, using the same flying monitoring system and under the same network conditions. The main results of field experiments aimed at comparing transmissions employing and not employing an external transceiver are:

- The ΔRSSI peaks (Figure 4), indicating a large improvement in RSSI, are due to the different slopes of the RSSI curves. In scenario S1, without an external transceiver, the RSSI curves are steeper. The addition of an external transceiver using beamforming made them longer and more gentle. The smallest RSSI improvement, 1 dBm, occurs at those distances where in both scenarios the operating point is on the flat, quasi-constant, section of the characteristic.
- The ΔADR peaks (Figure 6) are due to the delay between scenarios S1 and S2 in terms of ADR reduction. Off-peak, the increase in available data rate is relatively small, but the available data rate, i.e., the current maximum achievable data rate, only at the end of route A-A’ is less than the maximum total throughput. The last peak in Figure 6 refers to the reduction in ADR from an average of 26 Mbps to an average of just over 15 Mbps. In S1 this reduction took place at a distance of 95 m from point A, i.e., 5 m from point A’, in S2 at 100 m, i.e., at point A’.
- The total throughput is most influenced by the error rate because both data coming from sensors and video are congestion-controlled. Congestion control is triggered by transmission errors and even a small decrease in the error rate caused by small improvements in RSSI can result in large improvements in total throughput. This can be seen at the end of route A-A’, where the absolute improvement in total throughput, ΔThr, is 5.76 Mbps and the relative improvement in total throughput, δThr, is 61.46%.

From the analysis of the improvement in the RSSI curve, the improvement in the available data rate curve and the improvement in the total throughput curve, the following conclusions can be drawn:

- The external transceiver was able to extend the distance of error-free data and video transmission significantly by over 100%: from 25 m horizontally from the air station to the ground one to over 50 m.
- The external transceiver was able to significantly improve performance, measured in the RSSI and the available data rate, over short distances, i.e., less than 25 m. The use of an external transceiver over such a short distance may allow the air station carrying out the monitoring task to perform the additional transmission task, e.g., intermediating the transmission between the ground station and the next air station.
- The external transceiver improved the total throughput of transmission performed at a horizontal distance of 100 m from the ground station by 5.76 Mbps, i.e., over 60%. As a result, due to the use of an external transceiver, the total throughput of the aggregated stream (4K video and data from sensors) increased one and a half times: from about 10 Mbps to about 15 Mbps, which is still enough to assure trouble-free WebRTC transmission. Thus, the useful range, in which trouble-free data transmission from the UAV is still ensured, has increased from 85 m in scenario S1 to 100 m in scenario S2.

The presented results confirmed the usefulness of the external transceiver used in a wide range of transmission conditions. The benefit of employing an external transceiver always outweighed the cost of introducing an additional intermediate node on the air-to-ground section of the transmission path, even though this node, due to its unusual location, could not take advantage of the benefits resulting from shortening the air-to-ground distance. It was shown for all reported experiments the positive impact of using an external transceiver (indicated as a ΔRSSI value always greater than zero). The degree of this improvement depends on the distance between the air station and the ground one and is generally related to RSSI characteristics collected at the air station in both cases: when the WMMS was operating with and without the external transceiver.

Nowadays, WebRTC technology has a significant impact on media acquisition and enables the seamless transmission of media and non-media data over the Internet, so it seems to be a rather evident approach to use this technology in different use cases. In this paper, the case of UAV-based aggregated media and non-media transmission was reasonably defined and successfully implemented. Since this solution aims to reduce communication limitations, its use can be taken into account in UAV trajectory planning. Although the external transceiver was intended for applications with a small number of UAVs, this solution does not limit scalability. If a ground station without an external transceiver works with hundreds or thousands of nodes, then with an external transceiver it will work with them as well. Future research will focus on the impact of using different external transceivers and different network interfaces at the air station on the throughput achieved by WebRTC, as well as newer IEEE 802.11 family standards such as 802.11ax (Wi-Fi 6) and its successor Wi-Fi 7. The field experiment was performed in a real urban environment. Expanding the test to encompass various other circumstances also will be the subject of further research.

## Figures and Tables

**Table 1:** Related works.

Paper	Factor	Location	Basic Concepts	Intended Use
[2]	Interface	Both	Back-off and clustering for multiple UAV	EC ^1^ in disasters
[38]	Propagation	Air	Nodes of a chain network	Post-disaster EN ^2^
	Propagation	Ground	Nodes of a chain network	
[53]	Propagation	Air	Nodes of a chain network	GP ^3^
[37]	Antenna	Air	External antennas with a higher gain	GP ^3^
[35]	Antenna	Air	Vertical and horizontal polarization	GP ^3^
[34]	Antenna	Air	Directional antennas & automatic heading control	GP ^3^
	Propagation		Nodes of a chain network	
[21]	Antenna	Both	External antennas with a higher gain	GP ^3^
	Propagation	Air	Nodes of a mesh networks	EC ^1^ in disasters and SAR ^4^
[40]	Interference	Both	Beamforming in air-to-ground channel	GP ^3^
[41,42,43]	Interference	Ground	Beamforming implemented in SDR	GP ^3^
[39]	Interference	Ground	Explicit and implicit beamforming	GP ^3^
[49]	Propagation	Air	Nodes of a mesh network, range extender	EC ^1^ in disasters and SAR ^4^
[48]	Propagation	Ground	Range extender	GP ^3^
[50]	Propagation	Air	Nodes of a chain network	GP ^2^
			Nodes of a mesh network	
[51]	Propagation	Air	Nodes of a mesh network	EC ^1^ in disaster
[47]	Propagation	Ground	Nodes of ESS ^5^	EC ^1^ in disaster
[52]	Propagation	Air	Nodes of a mesh network	EC ^1^ for SAR ^4^
This paper	Interference	Ground	Beamforming in node of a chain network (external transceiver)	EC ^1^ in disasters and SAR ^4^

^1^ Emergency Communication; ^2^ Emergency Network; ^3^ General Purpose; ^4^ Search and Rescue; ^5^ Extended Service Set of IEEE 802.11 network.

**Table 2:** IEEE standards used in the papers listed in Table 1.

Paper	IEEE Standard	Comments
[2,41,42,43,48]	802.11	no detailed indication of the IEEE 802.11 standard
[35,38,40,50,51]	802.11n/ac	
[53]	802.11ah	
[37]	802.11g/n	
[34]	802.11b/g	
[21]	802.11a/n/ac 802.11s	single-hop: 802.11a, 802.11n, 802.11ac mesh: 802.11a + 802.11s
[39,47]	802.11ac	
[49,52]	802.11b	
This paper	802.11ac	

**Table 3:** Summary.

Paper	Method of Achieving the Improvement	Node Location	Access to the Area	Relative Cost
[38,49,50,51,52,53]	Using an intermediate node	Air	Not required	High
[38]	Using an intermediate node	Ground	Required	Low
[47]	Using an intermediate node	Ground	Not required	Low
[2,35,37,39,40,41,42,43]	Without using an intermediate node	Not applicable	Not required	High
[21,34]	Combined	Air	Not required	Low/High
[48]	Hybrid (range extender)	Ground	Not required	Low
This paper	Hybrid (external transceiver)	Ground	Not required	Low

**Table 4:** Air-to-ground transmission range extension for specific RSSI limits. The distances $d_{h}^{S1}$ and $d_{h}^{S2}$ are the largest horizontal distances between the ground station and the measurement point where the given limit has not yet been exceeded in the S1 and S2 scenarios, respectively.

Limit	$d_{h}^{S1}$	$d_{h}^{S2}$	$d_{h}^{S2}-d_{h}^{S1}$	$\frac{d_{h}^{S2}-d_{h}^{S1}}{d_{h}^{S1}}\cdot 100\%$	Comments
−67 dBm	25 m	45 m	20 m	80%	lossless real-time media streaming
−70 dBm	50 m	70 m	20 m	40%	lossless non-real-time data transmission
−80 dBm	85 m	90 m	5 m	5.9%	basic connectivity

**Table 5:** Extension of the transmission range at a given available data rate in the transition area and the second area, where: $mode_{ADR}$ is the available data rate mode, $d_{h}^{S1}$ and $d_{h}^{S2}$ are the horizontal distances between the ground station and the furthest measurement point where a given available data rate mode was calculated in scenario S1 and S2, respectively.

${\mathrm{mode}}_{\mathrm{ADR}}$	$d_{h}^{S1}$	$d_{h}^{S2}$	$d_{h}^{S2}-d_{h}^{S1}$	$\frac{d_{h}^{S2}-d_{h}^{S1}}{d_{h}^{S1}}\cdot 100\%$
60 Mbps	25 m	35 m	10 m	40%
54 Mbps	30 m	40 m	10 m	33.3%
26 Mbps	90 m	95 m	5 m	5.6%

## Data Availability

The data supporting the conclusions of this article will be made available by the authors on request.

## Abbreviations

The following abbreviations are used in this manuscript:

AP	Access Point
BSS	Basic Service Set
CCC	Command and Control Console
DiffServ	Differentiated Services
DS	Distribution System
DTLS	Datagram Transport Layer Security
ESC	Electronic Speed Control
ESS	Extended Service Set
EWS	Early Warning Systems
FAA	Federal Aviation Administration
FHSS	Frequency-Hopping Spread Spectrum
GNSS	Global Navigation Satellite System
GPIO	General Purpose Input-Output
HTML5	HyperText Markup Language version 5
IBSS	Independent BSS
ICE	Interactive Connectivity Establishment
IEEE	Institute of Electrical and Electronics Engineers
IoT	Internet of Things
IP	Internet Protocol
JSEP	JavaScript Session Establishment Protocol
LTE	Long Term Evolution
MQTT	Message Queue Telemetry Transport
MIMO	Multiple Input Multiple Output
NAT	Network Address Translation
QoS	Quality of Service
RTCP	Real-time Transport Control Protocol
RTP	Real-time Transport Protocol
RTP/SAVP	RTP Secure Audio/Video Profile
RSSI	Received Signal Strength Indicator
SAR	Search and Rescue
SBC	Single Board Computer
SCTP	Stream Control Transmission Protocol
SRTP	Secure RTP
SDP	Session Description Protocol
SIP	Session Initiation Protocol
STUN	Session Traversal Utilities for NAT
TCP	Transmission Control Protocol
TFRC	TCP-Friendly Rate Control
TURN	Traversal Using Relay NAT
UAV	Unmanned Aerial Vehicles
UDP	User Datagram Protocol
USB	Universal Serial Bus
W3C	World Wide Web Consortium
WebRTC	Web Real-Time Communications
WiMAX	Worldwide Interoperability for Microwave Access
WMMS	WebRTC Multimedia and Monitoring Station
WoT	Web of Things

## Indexes

The following indexes are used in this manuscript:

ADR	Refers to the available data rate
*h*	Horizontal
*k*	Measurement point number
S1	Scenario S1 (without external transceiver)
S1	Scenario S2 (with external transceiver)

## Symbols

The following symbols are used in this manuscript:

A	Beginning of the flight route (ground station location)
A’	End of flight route (100 m in a straight line from the point A)
ADR	Average available data rate value
*d*	Distance from point A (towards point A’)
Δ	Absolute improvement after using an external transceiver
δ	Relative improvement after using an external transceiver
mode	Modal value
RSSI	Average RSSI value
Thr	Average total throughput
